# Structure and belonging: Pathways to success for underrepresented minority and women PhD students in STEM fields

**DOI:** 10.1371/journal.pone.0209279

**Published:** 2019-01-09

**Authors:** Aaron J. Fisher, Rodolfo Mendoza-Denton, Colette Patt, Ira Young, Andrew Eppig, Robin L. Garrell, Douglas C. Rees, Tenea W. Nelson, Mark A. Richards

**Affiliations:** 1 University of California, Berkeley, Berkeley, CA., United States of America; 2 University of California, Los Angeles, Los Angeles, CA, United States of America; 3 California Institute of Technology, Los Angeles, CA, United States of America; 4 Stanford University’ Stanford, CA, United States of America; Indiana University, UNITED STATES

## Abstract

The advancement of underrepresented minority and women PhD students to elite postdoctoral and faculty positions in the STEM fields continues to lag that of majority males, despite decades of efforts to mitigate bias and increase opportunities for students from diverse backgrounds. In 2015, the National Science Foundation Alliance for Graduate Education and the Professoriate (NSF AGEP) California Alliance (Berkeley, Caltech, Stanford, UCLA) conducted a wide-ranging survey of graduate students across the mathematical, physical, engineering, and computer sciences in order to identify levers to improve the success of PhD students, and, in time, improve diversity in STEM leadership positions, especially the professoriate. The survey data were interpreted via path analysis, a method that identifies significant relationships, both direct and indirect, among various factors and outcomes of interest. We investigated two important outcomes: publication rates, which largely determine a new PhD student’s competitiveness in the academic marketplace, and subjective well-being. Women and minority students who perceived that they were well-prepared for their graduate courses and accepted by their colleagues (faculty and fellow students), and who experienced well-articulated and structured PhD programs, were most likely to publish at rates comparable to their male majority peers. Women PhD students experienced significantly higher levels of distress than their male peers, both majority and minority, while both women and minority student distress levels were mitigated by clearly-articulated expectations, perceiving that they were well-prepared for graduate level courses, and feeling accepted by their colleagues. It is unclear whether higher levels of distress in women students is related directly to their experiences in their STEM PhD programs. The findings suggest that mitigating factors that negatively affect diversity should not, in principle, require the investment of large resources, but rather requires attention to the local culture and structure of individual STEM PhD programs.

## Introduction

The underrepresentation of women and minorities in STEM fields continues to be a national concern as well as a priority for intervention in STEM education. Although women hold nearly 60% of faculty positions in psychology and 50% of these positions in the life sciences, women continue to hold less than 40% of STEM-related faculty positions, with even lower levels of representation in specific fields, such as physics and computer science. The representation of Black, Latinx, and American Indian/Alaska Native scholars in STEM fields, by comparison, remains at under 10% [1].

Although such disparities are vital to document and understand, they are likely to be poor targets for direct remediation. Attempting to mitigate the disparity itself (e.g., through attempts to increase enrollments) leaves untended the intermediate processes that may contribute to attrition along the path to the professoriate. Instead, interventions should endeavor to target the underlying mechanisms that lead to ethnic and gender-based disparities in STEM fields. By understanding the factors that confer additional risk and imbue resilience in women and underrepresented minorities, educators, administrators, and mentors might better address disparities in retention and job placement.

The present research is motivated by two parallel threads in the literature that address these disparities. The first line of work suggests that underrepresented students’ feelings of belonging within their academic programs may have far reaching effects on student performance [2]. For example, research has demonstrated that concerns about one’s lack of acceptance as a result of status characteristics (e.g., race, gender, social class, sexuality) can trigger psychological processes that negatively impact academic performance and persistence. More specifically, experiences of, or knowledge about exclusion or prejudice as a function of a given status characteristic can lead people who embody such characteristics to anxiously expect that they will be future targets of such treatment. These anxious expectations trigger increased attention for impending discrimination in threatening environments, which drains attentional resources away from academic tasks. Further, once perceived, the discrimination triggers strong emotional and physiological reactions, further disrupting performance [3]. Over time, these processes can lead to avoidance, disengagement, and disidentification from the threatening domain, as a coping mechanism against continued exposure to discrimination [4–6]. This process is then negatively-reinforced, as continued avoidance and disengagement is utilized to obviate and reduce discomfort.

Research on belonging uncertainty [7, 8] is consistent with these findings, documenting how feelings of social connectedness are particularly important for historically underrepresented individuals within the academy. For these individuals, cues about lack of belonging or reminders about their underrepresentation can trigger disruptive concerns about one’s own level of acceptance and belonging within the institution. Within STEM contexts, for example, the academic motivation of women is negatively affected by subtle but potent environmental cues that indicate lack of inclusivity, such as the far-off location of women's restrooms relative to men's [9], or a lack of representation of other women in the classroom [10]. In summary, then, independent research confirms that URM students' adjustment and academic outcomes in educational contexts in which they are the minority are directly affected by their concerns about belonging and about being the targets of discrimination within these very contexts.

The second line of research that informs the present work relates to the clarity of expectations and performance standards that characterizes the unit. Consistent with a literature suggesting that conditions of ambiguity are more likely to enable the expression of bias [11, 12], we have suggested that departmental structure within a graduate program—the degree to which programs have clear expectations, guidelines, and opportunities that are accessible to all students—may help account for performance disparities observed at the group level [13]. Consistent with this analysis, Mendoza-Denton et al. documented a case example of the College of Chemistry at UC Berkeley—an academic unit in which expectations and markers for progress are explicit, and students move through a structured training that is overseen by multiple individuals. Key to this department is that this “culture of structure” is embedded in the everyday activities of the community, such that the expectation to publish becomes the norm. Tellingly, Laursen and Weston [14] find that this unit is particularly successful in placing women PhD’s into academic positions, and our group found that publication rates in the college are comparable for women, URMs, and majority-group men–whereas significant disparities between these groups existed elsewhere. Clear expectations and structured programs may help counteract uneven treatment and the expression of negative stereotypes by encouraging professors to apply standards evenly across all students, by distributing knowledge about procedures and requirements thoroughly and uniformly, and by counteracting the effect of social cliques.

### The present research

Mendoza-Denton, Patt, and Richards [15] have proposed the two factors reviewed above–structure and belonging–as key dimensions along which academic units may differ, and which may contribute to the formation of inclusive environments that foster equitable participation across groups. Nevertheless, the relationship between belonging and structure remains unexamined. Whereas structure may facilitate performance-based outcomes, it is unknown whether it also mitigates differences in sense of belonging and well-being. Alternatively, structure and belonging may be orthogonal factors in predicting performance. Our research was motivated by a desire to understand the interrelationships between structure and belonging on academic outcomes in the real-world context of scholars enrolled at STEM departments across four highly competitive institutions. The collaborative effort of the four institutions allows us to examine pathways to success among a much larger group of students than would be possible at any one institution, while protecting the identities of students who may be severely underrepresented (and thus identifiable) in any one unit.

The doctoral programs represented here span four California universities: the University of California, Berkeley (UC Berkeley); the University of California, Los Angeles (UCLA); the California Institute of Technology (Caltech), and Stanford University. Although all are relatively close geographically, they represent both public and private institutions, and vary considerably in size. Further, these universities attract not only local students, but students from across the U.S. and the world, and place a disproportionate number of these students into the professoriate relative to other doctoral programs [16]. Together, then, the scholars at these four universities represent a broader and more geographically diverse sample than their geographical proximity might otherwise imply.

Structure was operationalized as the degree to which students perceived clear expectations and clear performance standards in their respective departments, and belonging was operationalized as the degree to which students felt accepted (positive valence) or insignificant (negative valence) in STEM settings. Subjective well-being was operationalized as the level of psychological and emotional distress participants reported. In order to account for precipitating conditions that may have contributed to *perceptions* of structure and belonging, we also accounted for the degree to which students felt prepared as advanced undergraduates, and the degree to which they felt prepared at the outset of graduate school.

Based on Mendoza-Denton et al. [13], we hypothesized that we would observe differential publication rates between majority male students, women, and URM students. Beyond these disparities, we were interested in delineating the causal pathways that account for these observed differences. In this way, we hoped to provide modifiable targets for academics and administrators, illuminating the functional connections between student perceptions of structure, feelings of belonging, and quantifiable and qualitative outcomes such as peer-reviewed publishing and well-being. The present research used path modeling to trace putative sequential chains of variables. Although we used cross-sectional data, the flow of information through the path models described below adheres to a logical temporal structure. That is, person-level characteristics such as race and gender precede variables related to undergraduate training, which precede variables related to graduate training, which are then followed by the outcomes of interest (subjective well-being and peer-reviewed publishing).

## Method

### Participants

Participants in this study consisted of graduate students in the National Science Foundation Alliance for Graduate Education and the Professoriate (NSF AGEP) California Alliance. The California Alliance encompasses UCLA, UC Berkeley, Stanford, and Caltech. The California Alliance focuses on increasing diversity in the academic fields with the greatest underrepresentation of minorities: the mathematical, physical, and computer sciences; and engineering (MPCS&E). The biological sciences are not included in this particular program. This study was authorized by the University of California, Berkeley institutional review board, approval #2013-10-5708. All participants completed informed consent.

Given the severe underrepresentation of minority scholars in STEM, we chose a recruitment strategy whereby all Black, Latinx, and American Indian/Alaska Native scholars were invited to participate in the study, as well as a randomly selected comparison sample of majority group students. This strategy allowed us to compare across groups, while also maintaining balance in the representation of participants. Tables 1 and 2 present the distributions of student demographics by field of study and institution, respectively. Across the four institutions, 499 students completed surveys: 114 students from Berkeley, 110 students from UCLA, 125 students from Stanford, and 150 students from Caltech. Females, both underrepresented minorities and non-underrepresented minorities, made up 221 of the students and 240 students were underrepresented minorities (47 black, 182 Latinx, and 11 Native American).

**Table 1:** Distribution of number (percentage) of female, black, and Latino students by field.

Percent	Female	Black	Latino	Male, Non-URM	Total
Engineering	94 (16.7%)	25 (4,4%)	105 (18.7%)	59 (10.5%0	283 (50.4%)
Chemistry	69 (12.3%)	7 (1.2%)	37 (6.6%)	19 (3.4%)	132 (23.5%)
Physics	17 (3.0%)	5 (1.0%)	18 (3.2%)	15 (2.7%)	55 (9.8%)
Earth and Planetary Science	18 (3.2%)	4 (0.7%)	7 (1.2%)	8 (1.4%)	37 (6.6%)
Other	23 (4.0%)	5 (0.9%)	15 (2.6%)	11 (2.0%)	54 (9.6%)
Total	221 (39.3%)	47 (8.4%)	182 (32.4%)	112 (19.9%)	N = 562

Note: Female category includes both URM and non-URM

**Table 2:** Distribution of number (percentage) of female, black, and Latino students by institution.

Percent	Female	Black	Latino	Male, Non-URM	Total
Berkeley	49 (8.7%)	17 (3.0%)	59 (10.5%)	17 (3.0%)	142 (25.3%)
UCLA	45 (8.0%)	7 (1.2%)	42 (7.4%)	26 (4.6%)	120 (21.3%)
Stanford	55 (9.8%)	14 (2.5%)	46 (8.2%)	25 (4.4%)	140 (24.9%)
Caltech	72 (12.8%)	9 (1.6%)	35 (6.2%)	44 7.8%)	160 (28.5%)
Total	221 (39.3%)	47 (8.4%)	182 (32.4%)	112 (19.9%)	N = 562

Note: Female category includes both URM and non-URM

### Measures

We utilized a survey instrument to assess students' experiences in graduate school, relationships with mentors and peers, progress in their doctoral program, and psychological factors. We utilized a survey instrument to assess students' experiences in graduate school, relationships with mentors and peers, progress in their doctoral program, and psychological factors, based on a broader literature on student adjustment in graduate programs [17–19]. Institutional records provide demographic data. For the present analyses, we focus on students’ (i) sense of belonging, (ii) their perceptions of departmental structure, (iii) subjective well-being, and (iv) publication success, as well as, (v) perceived level of preparation to account for background factors potentially affecting these variables. These assessments were embedded within a larger survey that we do not discuss here. The items–as presented to study participants–were worded as follows: (i) when I am in a science, technology, mathematics or engineering setting I feel accepted, and, when I am in a science, technology, mathematics or engineering setting I feel insignificant; (ii) the academic expectations of the department for graduate students are appropriate, and, the performance standards graduate students are held to are appropriate; (iii) feeling depressed, stressed, or upset; (iv) published in an academic journal; (v) when you took your first semester of graduate technical course work (in any area of math, science, or engineering) did you consider yourself, overall to be (less/as/more) prepared than the students in these classes, and, when you started taking advanced undergraduate technical courses for your major (in science, math, and/or engineering) did you consider yourself, overall, to be (less/as/more) prepared than the students in these classes. The survey materials, as presented to participants, can be found in Supplemental Materials.

The following demographic data also entered into a secondary model, described below, as covariates: general GRE and subject test scores at the time the respondent applied to graduate school; current graduate institution; academic discipline; years of doctoral study completed; remaining anticipated years of doctoral study; sex/gender, racial and ethnic identity; sexual orientation; health conditions that might impact learning, working or living activities; citizenship status; age; responsibility for dependents, and military service.

### Approach to path analysis

To address the questions of interest in the present study, we employed a path modeling approach. Originally derived by Wright in the early 20^th^ century [20–22], path modeling is a general linear model methodology that allows researchers to utilize two important extensions of regression analysis. First, in addition to assessing multiple predictor variables (as multiple regression does), path analysis can accommodate multiple *dependent* variables. Thus, multiple (potentially correlated) outcomes can be examined simultaneously. Second, path analysis allows variables to be both independent and dependent in a single analysis. That is, a single variable can be both a predictor and an outcome. Crucially, it is this aspect of the analysis that facilitates the examination of paths from a starting point to a downstream end point. In this way, we can examine both direct and indirect effects: that is, the effect of a predictor on an outcome (the direct effect), as well as the effect of a predictor as it travels through one or more intermediate variables on its way to the outcome (the indirect effect). In delineating pathways between gender and URM status and the *downstream* outcomes of interest (publication and subjective well-being), we can identify intermediate variables that confer increased risk or resilience within the graduate education process.

The proposed path models were tested within a structural equation modeling (SEM) framework, in a single-indicator path model in Mplus (version 7.2; [23]). That is, while no latent variables were modeled, the SEM framework was utilized to facilitate concurrent estimation of all direct and indirect relationships in the proposed models (a feature that is not possible with regression-based methods). Moreover, this approach allows the use of SEM fit statistics for evaluating the degree to which the model reflects the observed variance-covariance. Model fit was evaluated with the root mean square error of approximation (RMSEA), the chi-square goodness-of-fit test, and the confirmatory fit index (CFI). Non-significant chi-square tests, RMSEA values less than 0.060, and CFI values greater than or equal to 0.95 reflect excellent fit (see; [24]). The model provided continuous regression coefficients for all direct and indirect paths to subjective well-being, and a mixture of continuous and log-odds coefficients for predicting peer-reviewed publication. Log-odds coefficients are employed for any paths that terminate at a dichotomous outcome. Here, the peer-reviewed publication variable reflected whether or not each student had published in an academic journal. Gender and ethnicity are coded as independent variables in the analysis to variably describe participants; i.e., all participants are assigned a code for gender and a code for ethnicity), and are thus able to be entered as factors in the path analysis.

We used two data-driven approaches to selecting the final model. First, an initial model with only single-lag relationships was conducted. This initial model contained no direct effects that bypassed intermediate variables in the path model. We then used the Lagrange multiplier test–provided in Mplus as modification indices–to identify missing paths and direct relationships that were omitted in the initial model. Lagrange multiplier tests reveal chi-square changes associated with potential paths. The path with the largest associated chi-square change was added and the model was rerun. Additional paths were included until no significant modification indices remained (i.e. the addition of further paths would return a significant reduction in the model chi-square). Following the addition of paths via Lagrange multiplier, we then removed non-significant paths. This was likewise done one path at a time, starting with the smallest effects. Paths were removed until all remaining paths were significant at *p* < 0.05.

## Results

### Analytic sample

As we describe below, a central focus of our analyses was to identify potential discrepancies in the publication rate between URM and non-URM students. Of the URM sample, only 11 students were American-Indian/Native Alaskan. Given the small sample size, this group was excluded from inferential analyses, although we note that this small group nevertheless showed a very high percentage (7/11) who reported publishing in an academic journal. Additionally, to focus on a pool of students most likely to have published peer-reviewed publications, we restricted our analyses to those individuals who had completed required courses (per self-report). This removed 158 participants from analyses and left an effective sample size of 341. However, it should be emphasized that the comparison of publication rates and the path model described below yielded similar results when retested with the full sample.

### Publication rate

**Fig 1:**
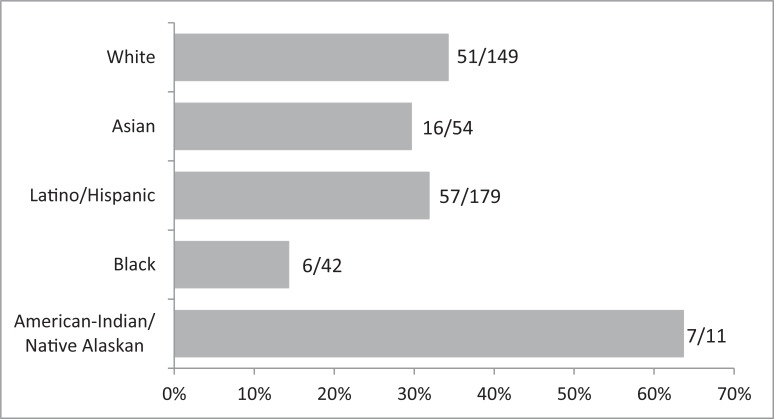
Percentage of students who have published a paper in an academic journal in the last year, by racial/ethnic/cultural designations.

We first examined differences in publication rate by ethnicity. Fig 1 presents the rates of publication by ethnic status. White, Asian, and Latinx students published in academic journals at roughly equivalent rates. Consistent with previous work from our group, Black graduate students appeared to publish at a rate significantly below their peers. This difference was statistically significant, with black students nearly three times less likely to have published a paper in an academic journal. Odds ratio (OR) for the sample who had completed required coursework was 2.74 (*z* = -2.14, *p* = 0.03), and the OR for the full sample was 2.82 (*z* = -2.46, *p* = 0.01).

### Test of proposed path model

We hypothesized that observed differences in publication rate would result from measurable discrepancies in students’ perceptions of structure and belonging. Specifically, given the discrepancy in publication rate between black students and their peers, the objective of the proposed path model was to find structural, explanatory paths that might account for this discrepancy. If the path model can mitigate the statistical discrepancy, then it can be hypothesized that the identified paths might reflect the mechanisms by which inequitable outcomes are generated. The path models described below had a temporal structure that flowed, left to right, from person-level characteristics (race and gender), to preparation for undergraduate classes, to preparation for graduate classes. The next temporal step included departmental expectations, departmental performance standards, feeling accepted in STEM settings, and feeling insignificant in STEM settings. These variables were then followed by perceived success, and, finally, the outcomes of interest (publication in an academic journal and subjective well-being).

**Fig 2:**
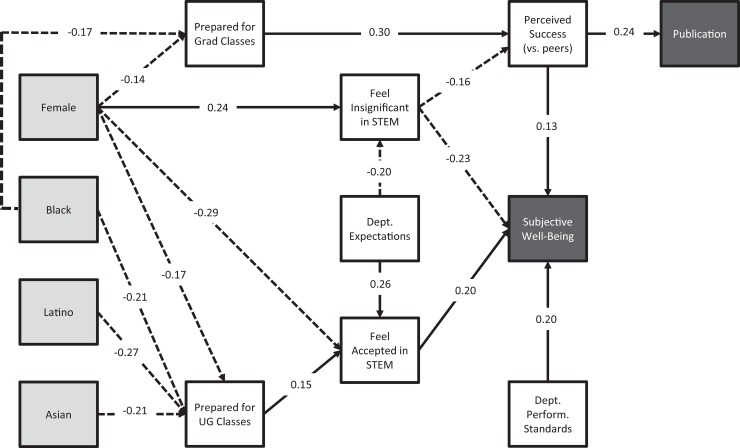
Final path model. Note: Dashed lines reflect negative relationship; solid lines reflect positive relationship; reference group = white male students.

Fig 2 presents the final path model for the relationship between race, gender, and downstream (i) likelihood to submit a peer-reviewed publication, and (ii) subjective well-being. Dotted paths indicate negative relationships and solid paths indicate positive relationships. Standardization puts coefficients on a scale from 1 to 1. Table 3 provides the standardized coefficients and accompanying SE’s, *t* and *p* values for the final model. The model provided an excellent fit to the data, χ2 (50) = 53.08, *p* = 0.36, RMSEA = 0.013, CFI = 0.99. The model accounted for 12% of the variance in likelihood to publish in an academic journal, 31% of the variance in subjective well-being, and 12% of the variance in perceived success relative to peers.

**Table 3:** Standardized coefficients, standard errors (S.E.s), t values, and p values for path model.

	Estimate	S.E.	t value	p value
**Prepared for advanced undergraduate classes**				
**Female**	-0.17	0.05	-3.17	0.002
**Black**	-0.21	0.05	-3.99	< 0.001
**Latino**	-0.27	0.06	-4.90	< 0.001
**Asian**	-0.21	0.06	-3.73	< 0.001
**Prepared for graduate classes**				
**Female**	-0.14	0.05	-2.77	0.006
**Black**	-0.17	0.05	-3.17	0.002
Feel accepted in STEM settings*				
**Female**	-0.29	0.06	-5.29	< 0.001
**Prepared UG**	0.15	0.05	3.00	0.003
**Dept. Expectations**	0.26	0.06	4.69	< 0.001
Feel insignificant in STEM settings*				
**Female**	0.24	0.06	4.07	< 0.001
**Dept. Expectations**	-0.20	0.06	-3.45	0.001
**Perceived success (relative to peers)**				
**Prepared Grad**	0.30	0.05	5.99	< 0.001
**Feel Insignificant**	-0.16	0.06	-2.85	0.004
**Psychological distress**^**+**^				
**Perceived Success**	-0.13	0.05	-2.52	0.012
**Female**	0.18	0.05	3.45	0.001
**Feel Accepted**	-0.20	0.06	-3.35	0.001
**Feel Insignificant**	0.23	0.06	3.72	< 0.001
**Dept. Standards**	-0.20	0.05	-3.97	< 0.001
**Submitted a peer-reviewed publication**^**+**^				
**Perceived Success**	0.24	0.05	4.66	< 0.001
**Black**	-0.08	0.05	-1.56	0.12

Note: Direction of temporal (i.e., predictive) order is from top to bottom

* and + indicate contemporaneous positions in path model

Prepared UG = degree to which respondent felt prepared for advanced undergraduate courses in their area; Prepared Grad = degree to which respondent felt prepared for graduate courses in their area; Dept. Expectations = degree to which respondent felt that there are clear expectations in their department; Dept. Standards = degree to which respondent felt that there are clear performance standards in their department.

The only *direct* predictor of publication was perceived success–greater levels of perceived success predicted a greater likelihood of publishing a manuscript. Female graduate students felt more insignificant in STEM settings and less prepared for graduate courses in their area of study. URM graduate students also perceived themselves as less prepared for graduate courses than their peers. Positive perceptions of departmental expectations reduced feelings of insignificance in STEM settings across all participants. The latter finding is consistent with our previous work with UC Berkeley STEM graduate students (Mendoza-Denton et al., 2017).

Overall, the model provided a robust picture of female graduate student perceptions of preparation, belonging, and departmental structure. Female students were more likely to feel insignificant in STEM settings, less likely to feel accepted in STEM settings, and perceived that they were less prepared for advanced undergraduate classes and graduate classes in their area of study. Feeling insignificant in STEM settings, in turn, led to lower perceived success and lower subjective well-being. Feeling accepted in STEM settings mitigated distress, as did positive perception of departmental performance standards. For URM students, paths to perceived success and subjective well-being led through perceived preparation, for both advanced undergraduate classes and graduate classes. URM students were less likely than their peers to perceive themselves to be prepared for coursework.

Finally, a model was run that included several potentially important control variables, including GRE scores, institution (with Berkeley as the reference) and progress toward degree (with dummy-coded variables for qualifying exams, preliminary exams, master’s degree, all but dissertation designation, and submitted dissertation). This model also provided an excellent fit to the data, χ2 (113) = 120.72, *p* = 0.29, RMSEA = 0.015, CFI = 0.98, and did not meaningfully affect the final model described above. The control model explained 15% of the variance in publication rate and 32% of the variance in subjective well-being.

## Discussion

Given previously identified disparities between the publication rates of majority males versus underrepresented minority (URM) and female graduate students (Mendoza-Denton et al., 2017), the present study focused on identifying the likelihood of publishing in an academic journal for majority and URM men and women. In addition to quantifying differences in published academic output between majority males and their female and URM peers, we were interested in determining the pathways that mediated individual subjective well-being, mindful that individual perceptions of quality of life will likely inform the pursuit of professional opportunities in STEM settings.

From extant research and theory, we identified a number of constructs likely to constitute pathways from student characteristics to both publication rate and subjective well-being. These included student beliefs that they were adequately prepared for advanced undergraduate classes, beliefs about preparation for graduate classes, perceptions of departmental expectations and standards, feeling accepted in STEM settings, feeling insignificant in STEM settings, and perceptions of success relative to peers. More broadly categorized, these variables reflected student preparation, departmental structure, and student perceptions of belonging.

To model the potentially complex interrelationships between these variables, we employed a path model approach, which presupposed temporal precedence among variables despite the cross-sectional nature of the data. The data have an implied temporal structure, with participants asked to report the degree to which they perceived being prepared as advanced undergrads and early graduate students. Because the present study leveraged this implied temporal structure, we used a subset of our student population who had completed all coursework: to wit, gender and race were assumed to precede all study variables. Preparation for undergraduate classes preceded preparation for graduate classes, which preceded current publication efforts and subjective well-being. Thus, we used the implied temporal structure of the data to model these relationships in a sequential path analysis, in order to test the direct and indirect effects of gender, race, preparation, structure, and belonging on success in publication and subjective well-being.

Preliminary analysis examined the relative distribution of academic publication rate across majority students (Asian and White) and URM students (disaggregated into Latino/Hispanic, Black, and Native American/Native Alaskan). The present study found that Latino/Hispanic and Native American/Alaskan students published in academic journals at rates at or above those of Asian and White students. However, Black students appeared to publish peer-reviewed papers at a significantly lower rate.

Path analyses targeting the contributions of perceived preparedness, structure and belonging to a dichotomous measure of having published a peer-reviewed paper mitigated the statistical significance of the discrepancy between black students and their peers. The model revealed one direct predictor and three indirect predictors of the likelihood of publishing in an academic journal. Perceptions of success directly predicted publication rates, whereas preparation for graduate classes, feeling insignificant in STEM settings, and perceptions of departmental expectations were all indirect predictors. Importantly, whereas we found that black students published at lower rates than their majority peers, perceived readiness, feelings of belonging and perceptions of program structure statistically mediated this link. That is, after accounting for these intermediate variables, there was no direct relationship between race and the likelihood to publish. This was true whether we modeled the overall sample or the subset of the sample that had completed required coursework, and whether or not we controlled for institution and progress toward degree.

The path analysis also examined factors that contribute to student’s subjective well-being, as measured by the degree to which students endorsed feeling depressed, stressed, or upset. Five direct predictors and five indirect predictors were identified. Gender, perceived success, feeling insignificant in STEM settings, feeling accepted in STEM settings, and perceptions of departmental performance standards each directly predicted subjective well-being. Feeling insignificant in STEM settings and being a woman both predicted increased distress, whereas feeling accepted in STEM settings, perceiving success relative to peers, and positive perceptions of performance standards all predicted increased well-being. Overall, this model accounted for 33% of the variance in subjective well-being, 16% of the variance in perceived success relative to peers, 19% of the variance in feeling accepted in STEM settings, and 11% of the variance in feeling insignificant in STEM settings.

Although a small, direct relationship existed between gender and subjective well-being, this path explained only 4% of the variance in subjective well-being (and no direct paths existed between minority categorizations and well-being). Given that the path model *en masse* predicted 33% of the variance in subjective well-being, the 4% directly predicted by gender represents only 12% of the total information the model explained about subjective well-being. Thus, 88% percent of the explained variance in subjective well-being came from intermediate pathways from preparation, belonging, and structure. Thus, we argue that these pathways and their constituent variables represent modifiable targets that could help to remediate the distress experienced by graduate students in STEM settings. To this end, the variable for departmental performance standards exhibited a direct, positive influence on subjective well-being–a modifiable factor that could affect *all* students.

### Limitations

It is important to note several limitations of this study. Importantly, the data from this study are cross-sectional, and thus any causal conclusions drawn here are necessarily assumptive, and should be considered preliminary. Nonetheless, as noted, there is an implied temporal structure to the variables (e.g., undergraduate preparation occurring prior to graduate experiences) that may reflect an underlying causal structure in the path analysis. Longitudinal analyses are needed to replicate these findings, perhaps even going so far as to intensively measure individuals on a student-by-student basis (see, 25]). Second, as noted, the sample is drawn exclusively from California universities. Concerns about this limitation, however, are mitigated by the fact that the students themselves are not exclusively from California: these programs draw highly qualified students from across the nation and the world. Nevertheless, the limitations associated with our findings suggest that future efforts replicating these findings should include a broader geographical range of institutions. Hopefully, such an effort would also help address issues of power, particularly as it pertains to American-Indian/Alaskan Native students.

## Conclusions

Despite the limitations noted above, the current dataset allows for a unique analysis of the mechanisms underlying disparities in fields where the number of underrepresented students can be vanishingly small. Our principal interest in this study was in understanding how group-level differences in student well-being and productivity might be explained by feelings of belonging and departmental structure, two variables that our prior research has identified as key in understanding achievement disparities. The findings reveal not only an effect of these variables, but importantly, a first picture of how these variables are interrelated with each other and with a select number of other important variables (e.g., sense of preparation). To summarize, gender and ethnic/racial inequities exist in levels of perceived preparation for the rigors of graduate school. These perceived inequities have a direct relationship to feelings of distress and belonging among students (which, in turn, may relate to feelings of impostorism that are often documented among underrepresented students in STEM fields; Tao & Gloria, 2018). Importantly, however, expectations and performance standards—what we conceptualize as structure—also have independent effects on acceptance, belonging, and subjective well-being, which then affect perceived success and publication rates.

The findings reported here seem to suggest a type of self-fulfilling cycle: students who feel less prepared in their undergraduate and graduate studies end up feeling less successful relative to their peers, and end up publishing less. Although it is tempting to see such a recursive process as being the fault and responsibility of the student, our analyses also make clear that student perceptions of departmental structures account for an appreciable amount of the variance in these processes. Doctoral programs are often highly unstructured learning and training environments, where individual autonomy and freedom are highly valued. Decisions as to what counts as a good idea, a worthwhile project, or adequate progress are often left to the discretion of professors, and criteria for success can be opaque for students. This is even more so for those who are not already “in the know.” Consistent with the findings highlighted here, in STEM disciplines, a perceived lack of acceptance and preparation may contribute to students not “leaning in” [26] in ways that facilitate or develop being in the know (Mendoza-Denton, in press). These findings support the notion that organizational interventions such as clarifying expectations and standards may help reduce academic disparities by potentially alleviating some of the distress associated with graduate education.

## Supporting information

S1 Data California alliance path model data. Note: id = deidentified participant number; courses = whether student completed required graduate courses; quals = whether student completed qualifying exams; masters = whether student completed master’s degree; abd = whether student is considered ‘all but dissertation;’ female = female biological sex; black, latino, white, asian, and urm denote membership is racial category; prepared1 = preparation for graduate classes; prepared2 = preparation for advanced undergraduate classes; success = perceived success, relative to peers; distress = feeling depressed, stressed, or upset; pub = whether student has published a peer-reviewed publication in the past year; accepted = degree to which student feels accepted in STEM settings; insignificant = degree to which student feels insignificant in STEM settings; expectations = perceptions of departmental expectations; standards = perceptions of departmental standards.
(XLSX)
